# Tumor heterogeneity and carcinoma in resected specimens of gastric low-grade dysplasia: A retrospective single center study

**DOI:** 10.1371/journal.pone.0280735

**Published:** 2023-01-25

**Authors:** Ga-Yeong Shin, Jun Young Park, Sung Hak Lee, Yu Kyung Cho, Myung-Gyu Choi, Jae Myung Park

**Affiliations:** 1 Division of Gastroenterology, Department of Internal Medicine, Seoul St. Mary’s Hospital, College of Medicine, The Catholic University of Korea, Seoul, South Korea; 2 Department of Clinical Pathology, Seoul St. Mary’s Hospital, The Catholic University of Korea College of Medicine, Seoul, South Korea; 3 Catholic Photomedicine Research Institute, The Catholic University of Korea, Seoul, South Korea; IRCCS Giovanni Paolo II Cancer Hospital, ITALY

## Abstract

Lesions diagnosed as gastric low-grade dysplasia (LGD) may be pathologically upgraded to early gastric cancer (EGC) or high-grade dysplasia (HGD) after endoscopic resection (ER). In this study, we investigated the risk factors for pathological upgrades after ER and assessed the reason for these upgrades by retrospectively analyzing ER data between January 1999 and December 2019. We enrolled patients with LGD confirmed by forceps biopsy; the patients were classified into pathologically concordant (LGD) and upgraded (HGD and EGC) groups according to the pathology of their resected specimen. To determine the risk factors for upgrade, we compared the endoscopic findings of the concordant and upgraded groups via 1:1 matched case-control design. To find the reasons for discordance, all upgraded cases were pathologically re-evaluated. Among 1,643 cases of LGD, pathological upgrades were observed in 423 (25.7%) resected specimens and EGC was found in 111 (6.7%) lesions. After matching the upgraded and concordant cases, lesion sizes exceeding 1.5 cm (odds ratio (OR): 1.8; 95% CI: 1.1–3.0), mucosal nodularity (OR: 10.8; 95% CI: 5.6–21.0), heterogeneous color (OR: 3.0; 95% CI: 1.7–5.3), presence of erosion (OR: 2.7; 95% CI: 1.8–5.3), and open-type gastric atrophy (OR: 2.9; 95% CI: 1.7–4.9) were noted to be significantly associated with upgraded pathology to EGC. Among the EGC cases, 99 (89.2%) were found to have pre-existing dysplasia. In conclusion, endoscopic evaluations should be performed because of possible pathological upgrades and co-existence of carcinomas in LGDs, especially when they exhibit surface nodularity, erosion, heterogeneous color, and large size.

## Introduction

According to the current multistep gastric carcinogenesis model, chronic inflammation of the gastric mucosa may progress to atrophic gastritis, intestinal metaplasia, dysplasia, and eventually gastric adenocarcinoma. The risk of such progression is dependent on the pathological grade of the dysplasia—the higher the grade, the higher is the risk [1, 2]. As such, pathological diagnoses of gastric neoplasia obtained via endoscopic forceps are important for the selection of appropriate therapeutic options. Many guidelines recommend endoscopic resection (ER) for the treatment of high-grade dysplasia (HGD) [3, 4]. However, the treatment of low-grade dysplasia (LGD) remains controversial in the current guidelines.

Gastric cancer has a poor prognosis in patients with advanced disease despite many improvements in understanding its pathogenesis [5–7]. Therefore, endoscopic examination is important for early diagnosis of gastric cancer. However, discrepancies in the pathological findings between forceps biopsies and resected specimens are common. In previous studies, 12–63% of forceps biopsies that confirmed LGDs were later upgraded to either HGD or early gastric cancer (EGC) [8–13]. These studies mainly focused on determining the risk factors for such upgrades by evaluating endoscopic factors, including lesion size and mucosal patterns. Most reports had limitations such as small sample size, lack of analysis for the influence of the environment around the tumor, including severity of atrophy, presence of *Helicobacter pylori* (*H*. *pylori*) infection, and multiplicity by synchronous lesions. Only a few studies have evaluated the reasons for such pathological discrepancies, especially for EGCs initially considered as gastric LSDs upon removal. Pathological upgrades are important because they can potentially lead to changes in the treatment strategy. In the present study, we retrospectively evaluated the causes and risk factors for pathological upgrades from gastric LGD to HGD or EGC after ER.

## Materials and methods

### Patients

We retrospectively analyzed the data of patients with gastric neoplasia who underwent ER between January 1999 and December 2019 at Seoul St. Mary’s Hospital (Seoul, Republic of Korea). Among them, 1,824 patients were initially diagnosed with LGD via endoscopic forceps biopsy. From this group, we sorted the patients who were ultimately diagnosed with adenoma and adenocarcinoma based on pathological reviews of the resected specimens. We excluded cases of inflammation (n = 146), other synchronous lesions (n = 12), hyperplastic polyps (n = 18), and poorly differentiated carcinomas (n = 4) in the resected specimens. We then collected data, including demographic characteristics, pathology, endoscopy results, and *H*. *pylori* status from the electronic patient charts. We define a synchronous lesion as a lesion whose appearance did not vary 12 months after the diagnosis of the initial main neoplasm. The Institutional Review Board of the study institution approved this study and waived patients consent (IRB number KC20RISI1022).

### Endoscopic evaluation and procedures

We reviewed the endoscopic reports to evaluate the features of the discovered lesions. At the time of ER, the extent of gastric atrophy was determined by endoscopic findings and categorized as closed- or open-type based on the Kimura–Takemoto classification [14]. Endoscopic photographs were reviewed in all cases by three endoscopists (GYS, JMP, and CHL), all of whom have extensive experience, with over 10,000 diagnostic examinations and ERs. All reviews were performed in a blinded manner. The location of the lesion was described using the Japanese Classification of Gastric Cancer [15]. The Paris Classification was used to define the gross type of the superficial lesion, which was categorized as elevated, flat, or depressed [16]. Lesions with both elevated and depressed areas were classified into the depressed group. We evaluated the characteristics of the mucosal surfaces according to nodularity, heterogeneous color, presence of erosion, and spontaneous bleeding. In this work, nodularity was defined as the presence of irregularly raised or nodular mucosa [17]; heterogenous color was defined as discoloration of the mucosal surface of the lesion compared to the surrounding mucosa; erosion was defined as a flat or slightly depressed mucosal break of less than 5 mm diameter, with the mucosal defects limited to the mucosa [18]; spontaneous bleeding was defined as bleeding before the forceps biopsy or bleeding from a weak touch.

Indigo carmine solution was sprayed on the gastric mucosa to mark the lesion before making a circumferential incision. A snare was used in the endoscopic mucosal resection (EMR), and a Hook knife (Olympus Medical Systems Co. Ltd., Tokyo, Japan) was used in the endoscopic submucosal dissection (ESD). Simultaneous electronic coagulation was applied during ER; all ER procedures were performed by expert endoscopists with more than five years of experience each.

### Pathological evaluations

The resected specimens were stretched, pinned, and fixed with formalin. Piecemeal-resected specimens were then reconstructed to their original shapes as much as possible. The fixed specimens were transversely sectioned at 2 mm intervals. Five-micrometer sections were then routinely cut and stained with hematoxylin and eosin. The pathological diagnoses were based on the revised Vienna classification [19] and were made on the basis of the Japanese Classification of Gastric Carcinoma, which included the size, histological type, and depth of invasion [15]. All endoscopically resected tissues were reviewed by two GI pathologists (IHS and SHL). In the pathologically upgraded cases, the original endoscopic forceps biopsy specimens and resected specimens were re-evaluated in a blinded manner to determine the cause of discrepancy in the diagnosis. *H*. *pylori* infection was diagnosed based on a rapid urease test (CLO^TM^ test, Ballard Medical Products, Draper, UT, USA), DPO-based multiplex polymerase chain reaction analysis (Seeplex® ClaR–*H*. *pylori* ACE Detection kit, Seegene Inc., Seoul, Korea), or histological examination by Warthin–Starry silver staining at the time of ER.

### Statistical analysis

To determine the risk factors for the pathological upgrades, we evaluated the data using 1:1 matched case-control design. The cases were defined as patients in whom the LGD confirmed by biopsy was subsequently pathologically upgraded after ER (upgrade group). The controls were the patients in whom the pathological diagnoses were concordant before and after ER (concordant group); the control group was matched 1:1 with the case group by age and sex at the time of diagnosis. Univariate analysis was performed with the chi-squared test or Fisher’s exact test for the categorical variables or Student’s t-test for the continuous variables. Variables with *p* < 0.05 in the univariate analyses were included in a forward stepwise multiple logistic regression model to identify the independently associated risk factors of early gastric cancer. A value of *p* < 0.05 was considered to be statistically significant. The statistical analyses were performed with SPSS version 21.0 for Windows (SPSS Inc, Chicago, IL, USA).

## Results

### Baseline characteristics of the patients

**Fig 1:**
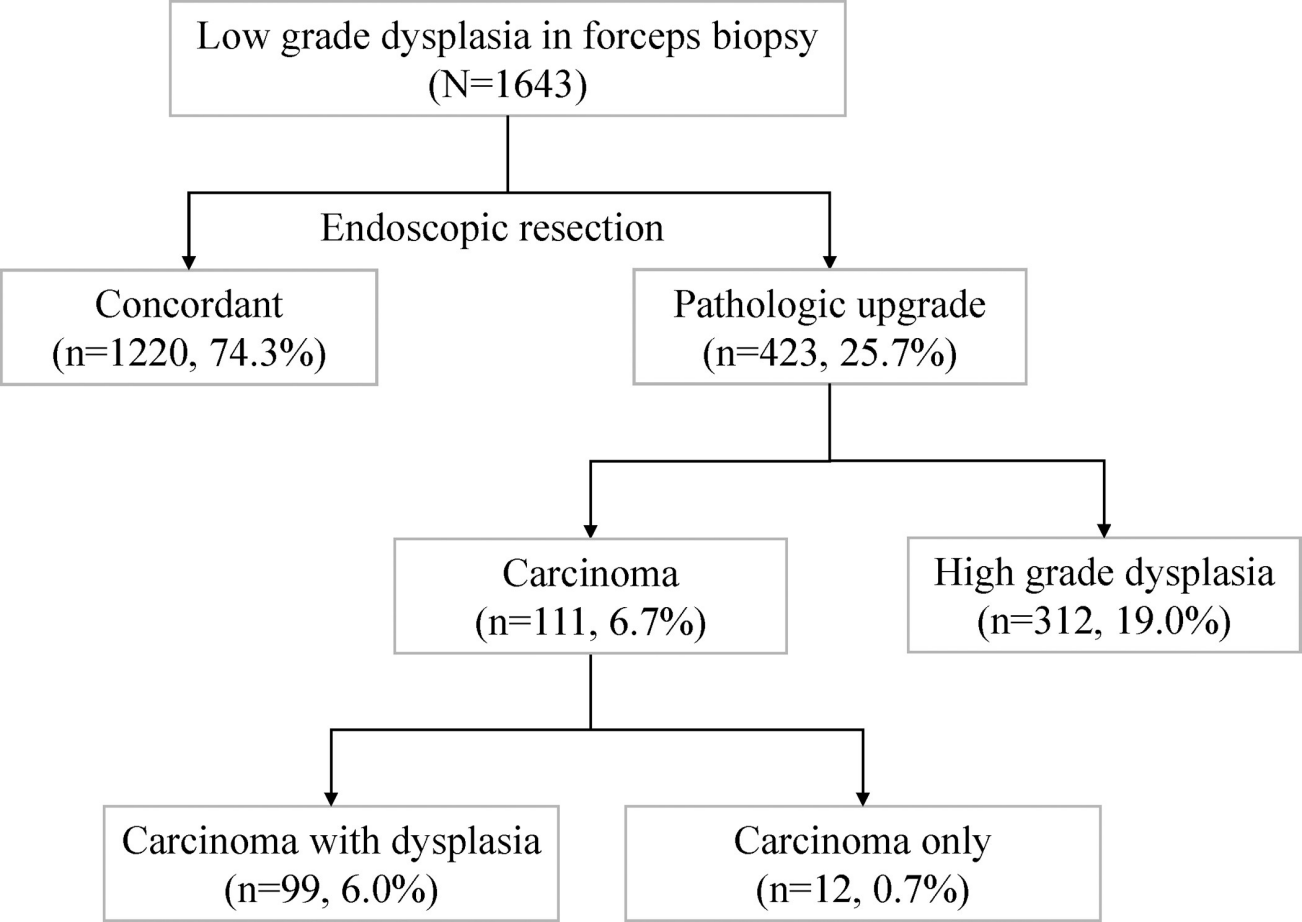
Pathologies of the endoscopic forceps biopsies and resected specimens.

A total of 1,643 gastric LGDs were analyzed in this study. After ER, pathological upgrades were made to 423 (25.7%) cases: 111 (6.8%) cases of EGCs and 312 (19.0%) cases of HGDs (Fig 1). Based on the age and sex, the pathologically upgraded cases were 1:1 matched with the control group. The mean age was 64 years, and males comprised about 70% of the cases (Table 1). The baseline characteristics of the cases and controls are summarized in Table 1.

**Table 1:** Clinical and endoscopic characteristics of the pathologically concordant and upgraded groups after resection of gastric low-grade dysplasia.

		Total (N = 846)	Pathology after endoscopic resection		Univariate analysis		Multivariate analysis	
			Concordant (n = 423)	Upgrade (n = 423)	Odds ratio (95% CI)	*P*	Odds ratio (95% CI)	*P*
Age (mean ± SD)		64.1 ± 8.9	64.1 ± 8.9	64.1 ± 9.0				
Sex								
	Male	586 (69.3%)	293 (69.3%)	293 (69.3%)				
	Female	260 (30.7%)	130 (30.7%)	130 (30.7%)				
Location (n, %)								
	Lower	548 (64.8%)	273 (64.5%)	275 (65.0%)	Reference			
	Middle	239 (28.2%)	117 (27.7%)	122 (28.8%)	1.03 (0.76–1.40)	0.87		
	Upper	59 (7.0%)	33 (7.8%)	26 (6.1%)	0.76 (0.44–1.30)	0.32		
Gross type (n, %)								
	Elevated	107 (12.6%)	62 (14.7%)	45 (10.6%)	Reference			
	Flat	658 (77.8%)	324 (76.6%)	334 (79.0%)	1.44 (0.95–2.17)	0.08		
	Depressed*	81 (9.6%)	37 (8.7%)	44 (10.4%)	1.67 (0.93–2.98)	0.09		
Lesion size (n, %)								
	<1.5 cm	531 (62.8%)	305 (72.1%)	226 (53.4%)	Reference		Reference	
	≥1.5 cm	315 (37.2%)	118 (27.9%)	197 (46.6%)	2.27 (1.76–3.02)	<0.01	1.88 (1.39–2.54)	<0.01
Macroscopic pattern (n, %)								
	Nodularity	389 (46.0%)	137 (32.4%)	252 (59.6%)	3.11 (2.34–4.12)	<0.01	2.32 (1.72–3.15)	<0.01
	Heterogeneous color	236 (27.9%)	84 (19.9%)	152 (35.9%)	2.28 (1.67–3.11)	<0.01	1.38 (0.97–1.97)	0.08
	Erosion	64 (7.6%)	12 (2.8%)	52 (12.3%)	4.83 (2.54–9.18)	<0.01	2.81 (1.39–5.71)	<0.01
	Spontaneous bleeding	7 (0.8%)	1 (0.2%)	6 (1.4%)	6.10 (0.73–50.9)	0.10		
Atrophy (n, %)								
	Closed type	504 (59.6%)	275 (65.0%)	229 (54.1%)	Reference		Reference	
	Open type	342 (40.4%)	148 (35.0%)	194 (45.9%)	1.56 (1.18–2.05)	0.002	1.17 (0.87–1.58)	0.31
*Helicobacter pylori* infection (n, %)		406 (48.0%)	203 (48.0%)	203 (48.0%)	0.92 (0.69–1.22)	0.557		
Synchronous lesion (n, %)		140 (32.0%)	70 (16.5%)	70 (16.5%)	1.24 (0.87–1.76)	0.233		

* Lesions with both elevated and depressed areas were classified as depressed.

### Risk factors associated with pathological upgrades after resection

The clinicopathological characteristics of the pathologically concordant and upgraded groups are shown in Table 1. The lesion size, nodularity, heterogeneous color, erosion, spontaneous bleeding, and atrophy were significantly different between the concordant and upgrade groups. In univariate analysis, a lesion size greater than 1.5 cm (OR: 2.27; 95% CI: 1.76–3.02), mucosal nodularity (OR: 3.11; 95% CI: 2.34–4.12), heterogeneous color (OR: 2.28; 95% CI: 1.67–3.11), presence of erosion (OR: 4.83; 95% CI: 2.54–9.18) within the lesion, and open-type gastric atrophy (OR: 1.56; 95% CI: 1.18–2.05) were all significantly associated with upgraded pathology. In multivariate analysis, a lesion size greater than 1.5 cm (OR: 1.88; 95% CI: 1.39–2.54), mucosal nodularity (OR: 2.32; 95% CI: 1.72–3.15), and presence of erosion (OR: 2.81; 95% CI: 1.39–5.71) within the lesion were all significantly associated with upgraded pathology (Table 1).

### Risk factors for pathological upgrade to EGC

When we compared the clinicopathological characteristics of the concordant group with those of the upgrade group whose diagnosis was later revised to EGC, a lesion size greater than 1.5 cm (OR: 2.94; 95% CI: 1.92–4.52), mucosal nodularity (OR: 17.28; 95% CI: 9.18–32.54), heterogeneous color (OR: 5.31; 95% CI: 3.40–8.29), presence of erosion (OR: 10.50; 95% CI: 5.10–21.64), spontaneous bleeding (OR: 11.75; 95% CI: 1.21–114.08) in the lesion, and open-type gastric atrophy (OR: 3.85; 95% CI: 2.47–6.00) were all significantly associated with histopathological upgrade based on the univariate analysis. In the multivariate analysis, a lesion size greater than 1.5 cm (OR: 1.80; 95% CI: 1.06–3.04), mucosal nodularity (OR: 10.80; 95% CI: 5.55–20.97), heterogeneous color (OR: 3.02; 95% CI: 1.73–5.31), presence of erosion (OR: 2.69; 95% CI: 1.75–5.31), and open-type gastric atrophy (OR: 2.89; 95% CI: 1.69–4.91) were significantly associated with pathological upgrade to EGC (Table 2).

**Table 2:** Risk factors associated with histological upgrade to early gastric cancer.

		Concordant group (n = 423)	Cancer upgrade group (n = 111)	Univariate analysis		Multivariate analysis	
				Odds ratio (95% CI)	P	Odds ratio (95% CI)	P
Age (mean ± SD)		64.1 ± 8.9	64.1 ± 8.9				
Sex							
	Male	293 (69.3%)	80 (72.1%)				
	Female	130 (30.7%)	31 (27.9%)				
Location (n, %)							
	Lower	273 (64.5%)	70 (63.1%)	Reference			
	Middle	117 (27.7%)	32 (28.8%)	1.07 (0.67–1.71)	0.79		
	Upper	33 (7.8%)	9 (8.1%)	1.03 (0.47–2.26)	0.94		
Gross type (n, %)							
	Elevated	62 (14.7%)	14 (12.6%)	Reference			
	Flat	324 (76.6%)	86 (77.5%)	1.19 (0.64–2.23)	0.58		
	Depressed	37 (8.7%)	11 (9.9%)	1.34 (0.55–3.25)	0.52		
Lesion size (cm)							
	<1.5	305 (72.1%)	52 (46.8%)	Reference		Reference	
	≥1.5	118 (27.9%)	59 (53.2%)	2.94 (1.92–4.52)	<0.01	1.80 (1.06–3.04)	0.03
Macroscopic pattern							
	Nodularity	137 (32.4%)	99 (89.2%)	17.28 (9.18–32.54)	<0.01	10.80 (5.55–20.97)	<0.01
	Heterogeneous color	84 (19.9%)	63 (56.8%)	5.31 (3.40–8.29)	<0.01	3.02 (1.73–5.31)	<0.01
	Erosion	12 (2.8%)	26 (23.4%)	10.50 (5.10–21.64)	<0.01	2.69 (1.10–6.56)	0.03
	Spontaneous bleeding	1 (0.2%)	3 (2.7%)	11.75 (1.21–114.08)	0.03	3.40 (0.13–88.76)	0.46
Atrophy							
	Closed type	275 (65.0%)	36 (32.4%)	Reference		Reference	
	Open type	148 (35.0%)	75 (67.6%)	3.85 (2.47–6.00)	<0.01	2.89 (1.69–4.91)	<0.01
*Helicobacter pylori* infection (n, %)		203 (48.0%)	56 (50.5%)	1.19 (0.77–1.82)	0.43		
Synchronous lesion		70 (16.5%)	22 (19.8%)	1.25 (0.73–2.13)	0.411		

### Reasons for discordant pathology

Endoscopic biopsies and resected specimens were pathologically re-evaluated to determine the reasons for discordance in the EGC cases. We found that 99 (89.2%) of the 111 EGCs developed in settings with pre-existing dysplasia. Among them, only one case was found to have carcinoma in the background of LGD and others in the background of HGD in the resected specimens. Twelve cases (10.8%) were found with atypia in the endoscopic biopsies, which made it impossible to arrive at definitive diagnoses by endoscopic biopsy.

## Discussion

In the present study, we observed pathological upgrades in about 25% of the LGD cases. Among the upgrades, EGC comprised about one-fourth of the cases. In total, about 7% of the cases that were initially considered to be LGD were pathologically confirmed as carcinoma from the resected specimens. The main reason for such a discrepancy was the heterogenicity of EGCs arising from pre-existing adenomas. The risk factors associated with pathological upgrade to EGC include large size, macroscopic morphology with mucosal nodularity, heterogeneous color, presence of erosion in the lesion, and open-type gastric atrophy. Interestingly, about 1% of cases were EGCs based on the resected specimens, for which diagnosis by endoscopic forceps biopsy was not possible.

The clinical criteria for the management of gastric LGD are shrouded in controversy because of its relatively low risk of progressing to EGC. However, the present study showed that pathological discrepancies between forceps biopsies and resected specimens are more common than thought. In a meta-analysis, one out of four LGD pretreatment biopsies were considered to have been misdiagnosed and were subsequently pathologically upgraded to HGD or EGC. This finding is consistent with the results of our study [20]. Therefore, gastric biopsy should not be used for a final diagnosis in the case of LGD; the lesions must be resected and fully pathologically reviewed to verify the initial diagnosis. In particular, the present study as well as previous results showed that macroscopic inhomogeneities and mucosal friability should be considered as risk factors for the pathological upgrades. When these findings are observed with pathological LGDs, endoscopists should consider the possibility of carcinomatous changes in the lesions.

There are several plausible reasons for why some dysplasias are upgraded to cancer. In general, there may be individual differences in the diagnostic criteria for distinguishing similar yet different lesions. Moreover, LGD and regenerative atypia or LGD and HGD may be perceived differently by different pathologists. Another reason for the upgraded pathology can be the diagnostic inaccuracy of the endoscopic biopsy. Our pathologists have consistently reported such cases as “atypia”. Therefore, more accurate selection of the biopsy site and adequate numbers of samples can help reduce diagnostic inaccuracies. In addition, there may be a sample size problem; it is possible that a finding of HGD or adenocarcinoma from a pathological review of the resected specimen may be initially misdiagnosed as LGD based on the endoscopic biopsy owing to the limited depiction of the entire lesion.

Prior studies have also evaluated the effects of heterogeneity, especially the possibility of adenocarcinoma, in the pathological discrepancies [21, 22]. We aimed to determine the reason for such diagnostic discrepancies by reviewing the EGC cases. In the present study, we found that most diagnostic discrepancies involving EGCs were attributable to tumor heterogeneity. We also found that such heterogeneities may be indirectly suspected when endoscopic findings like mucosal nodularity, uneven color, presence of erosion, and spontaneous bleeding are present in the lesion. Therefore, the endoscopic biopsy specimen may not be representative of the entire lesion in such cases mostly owing to tumor heterogeneities. Our data support the evidence of the multistep gastric carcinoma genesis model [1].

Previous studies have evaluated the risk factors for pathological upgrades after ER, which may be associated with the heterogenicity of EGCs [8–13]. These studies reported that large-sized lesions, usually greater than 1.5 cm, were risk factors for EGC in adenomas. This correlation suggests that size may be proportional to disease progression. Endoscopic findings such as erythema, redness, and depressed lesions have been reported to be associated with potential pathological upgrades [8–13]; this indicates that microscopic structural changes during disease progression may be reflected in the macroscopic findings [21, 23]. These factors were consistently observed in the present study as well. We also evaluated whether open-type atrophy was specifically associated with histopathological upgrade to EGC. It is known that the severity of atrophic gastritis increases the risk of gastric cancer [24, 25]. However, *H*. *pylori* infections were not significantly associated; *H*. *pylori* has been linked to chronic atrophic gastritis, an established precursor of the intestinal type of gastric carcinoma. The model known as the Correa cascade proposes the steps of disease progression, with *H*. *pylori* infection as an etiological factor in each step of the cascade [1, 26]. In our studies, the main reason for the pathological discrepancy was that the cancers originated from pre-existing adenomas. This means that the risk factors in the pathological discrepancies may be associated with the risk of gastric cancers originating from pre-existing adenomas.

To overcome the pathological discrepancies in gastric forceps biopsy, endoscopists should consider the heterogenicity of the lesion as well as the risk factors, including lesion size, macroscopic patterns including nodularity, erosion, heterogeneous color, and open-type atrophic gastric mucosa. The most important thing is to perform the biopsy at the target lesion. First, it is important to perform high-quality esophagogastroduodenoscopy (EGD) [27]. In addition, adequate observation time during the EGD would be helpful for minimizing blind spots when evaluating the risk factors of the lesion [28]. Other endoscopic imaging techniques, such as ME-NBI and IEE, have been reported to show high accuracies for evaluating the histological characteristics of EGCs and histological severity of gastritis. Adoption of such techniques during the procedure would thus be helpful in reducing pathologic discrepancies as well [8, 29]. Finally, however, it would be helpful to endoscopically resect the lesions confirmed as low-grade adenomas in pretreatment biopsies for appropriate diagnosis and treatment.

This study has several strengths. First, we were able to evaluate more than 1,500 cases of resected specimens. Second, two GI-specialty pathologists reviewed all tissues that were pathologically upgraded from LGD to EGC. Therefore, we tried to exclude pathological interobserver variations. Third, the endoscopic images were also reviewed by three endoscopy specialists for the same reason.

This study also has several limitations. First, this was a retrospective 1:1 matched case-control design, so patient selection bias may have been present. Second, our review of endoscopic biopsies was incomplete for patients who were referred from primary clinics since many of them only brought written pathology reports and not specimen slides. Therefore, the cause of upgraded pathology to HGD may have been from interobserver variations among pathologists [30]. However, the present study showed that the overall concordance of the diagnosis of gastric dysplasia and early carcinoma in ESD specimens was excellent, which indirectly indicates that interobserver variations among pathologists may be low. Furthermore, all EGC cases were re-reviewed for both the endoscopic biopsies and resected specimens. Lastly, this study was performed in a single center.

In this study, we showed that LGD is also at risk for gastric cancer in a large number of endoscopic resection specimens. For a more robust analysis, we reviewed all resected specimens again by two pathologists. From this data, gastric LGD should be considered for complete resection owing to the possibility of cancers arising from pre-existing adenomatous lesions with surface nodularities, mucosal erosion, sizes greater than 1.5 cm, heterogeneous color, and background mucosa with open-type atrophy. Endoscopists should thus pay attention to evaluating the presence of these risk factors for the possibility of carcinogenesis in the lesions. We believe that artificial intelligence or digital pathology using endoscopy can solve tumor heterogeneity in the future.

## Supporting information

S1 Data (XLSX)

## References

[1] Correa P. Human gastric carcinogenesis: a multistep and multifactorial process—First American Cancer Society Award Lecture on Cancer Epidemiology and Prevention. Cancer Res. 1992;52(24):6735–40. Epub 1992/12/15. . 1458460

[2] de Vries AC, van Grieken NC, Looman CW, Casparie MK, de Vries E, Meijer GA, et al. Gastric cancer risk in patients with premalignant gastric lesions: a nationwide cohort study in the Netherlands. Gastroenterology. 2008;134(4):945–52. Epub 2008/04/09. doi: 10.1053/j.gastro.2008.01.071 . 18395075

[3] Pimentel-Nunes P, Libânio D, Marcos-Pinto R, Areia M, Leja M, Esposito G, et al. Management of epithelial precancerous conditions and lesions in the stomach (MAPS II): European Society of Gastrointestinal Endoscopy (ESGE), European Helicobacter and Microbiota Study Group (EHMSG), European Society of Pathology (ESP), and Sociedade Portuguesa de Endoscopia Digestiva (SPED) guideline update 2019. Endoscopy. 2019;51(04):365–88. Epub 06.03.2019. doi: 10.1055/a-0859-1883 30841008

[4] Banks M, Graham D, Jansen M, Gotoda T, Coda S, di Pietro M, et al. British Society of Gastroenterology guidelines on the diagnosis and management of patients at risk of gastric adenocarcinoma. Gut. 2019;68(9):1545. doi: 10.1136/gutjnl-2018-318126 31278206 PMC6709778

[5] Ricci AD, Rizzo A, Brandi G. DNA damage response alterations in gastric cancer: knocking down a new wall. Future Oncol. 2021;17(8):865–8. Epub 2021/01/30. doi: 10.2217/fon-2020-0989 . 33508962

[6] Ricci AD, Rizzo A, Rojas Llimpe FL, Di Fabio F, De Biase D, Rihawi K. Novel HER2-Directed Treatments in Advanced Gastric Carcinoma: AnotHER Paradigm Shift? Cancers (Basel). 2021;13(7). Epub 2021/05/01. doi: 10.3390/cancers13071664 ; PubMed Central PMCID: PMC8036476. 33916206 PMC8036476

[7] Rihawi K, Ricci AD, Rizzo A, Brocchi S, Marasco G, Pastore LV, et al. Tumor-Associated Macrophages and Inflammatory Microenvironment in Gastric Cancer: Novel Translational Implications. Int J Mol Sci. 2021;22(8). Epub 2021/05/01. doi: 10.3390/ijms22083805 ; PubMed Central PMCID: PMC8067563. 33916915 PMC8067563

[8] Yang L, Jin P, Wang X, Zhang T, He YQ, Zhao XJ, et al. Risk factors associated with histological upgrade of gastric low-grade dysplasia on pretreatment biopsy. J Dig Dis. 2018;19(10):596–604. Epub 2018/09/07. doi: 10.1111/1751-2980.12669 . 30187683

[9] Ryu DG, Choi CW, Kang DH, Kim HW, Park SB, Kim SJ, et al. Pathologic outcomes of endoscopic submucosal dissection for gastric epithelial neoplasia. Medicine (Baltimore). 2018;97(33):e11802. Epub 2018/08/17. doi: 10.1097/MD.0000000000011802 ; PubMed Central PMCID: PMC6112879. 30113468 PMC6112879

[10] Maekawa A, Kato M, Nakamura T, Komori M, Yamada T, Yamamoto K, et al. Incidence of gastric adenocarcinoma among lesions diagnosed as low-grade adenoma/dysplasia on endoscopic biopsy: A multicenter, prospective, observational study. Dig Endosc. 2018;30(2):228–35. Epub 2017/11/03. doi: 10.1111/den.12980 . 29094455

[11] Kang DH, Choi CW, Kim HW, Park SB, Kim SJ, Nam HS, et al. Predictors of upstage diagnosis after endoscopic resection of gastric low-grade dysplasia. Surg Endosc. 2018;32(6):2732–8. Epub 2017/12/08. doi: 10.1007/s00464-017-5971-5 . 29214514

[12] Xu G, Zhang W, Lv Y, Zhang B, Sun Q, Ling T, et al. Risk factors for under-diagnosis of gastric intraepithelial neoplasia and early gastric carcinoma in endoscopic forceps biopsy in comparison with endoscopic submucosal dissection in Chinese patients. Surg Endosc. 2016;30(7):2716–22. Epub 2015/10/02. doi: 10.1007/s00464-015-4534-x . 26423416

[13] Cho SJ, Choi IJ, Kim CG, Lee JY, Kook MC, Park S, et al. Risk of high-grade dysplasia or carcinoma in gastric biopsy-proven low-grade dysplasia: an analysis using the Vienna classification. Endoscopy. 2011;43(6):465–71. Epub 2011/03/23. doi: 10.1055/s-0030-1256236 . 21425043

[14] Kimura K, Takemoto T. An Endoscopic Recognition of the Atrophic Border and its Significance in Chronic Gastritis. Endoscopy. 1969;1(03):87–97. doi: 10.1055/s-0028-1098086

[15] Japanese Gastric Cancer A. Japanese classification of gastric carcinoma: 3rd English edition. Gastric Cancer. 2011;14(2):101–12. Epub 2011/05/17. doi: 10.1007/s10120-011-0041-5 . 21573743

[16] The Paris endoscopic classification of superficial neoplastic lesions: esophagus, stomach, and colon: November 30 to December 1, 2002. Gastrointest Endosc. 2003;58(6 Suppl):S3–43. Epub 2003/12/04. doi: 10.1016/s0016-5107(03)02159-x . 14652541

[17] Jung SJ, Cho SJ, Choi IJ, Kook MC, Kim CG, Lee JY, et al. Argon plasma coagulation is safe and effective for treating smaller gastric lesions with low-grade dysplasia: a comparison with endoscopic submucosal dissection. Surg Endosc. 2013;27(4):1211–8. Epub 2012/10/19. doi: 10.1007/s00464-012-2577-9 . 23076459

[18] Yamamoto S, Watabe K, Tsutsui S, Kiso S, Hamasaki T, Kato M, et al. Lower serum level of adiponectin is associated with increased risk of endoscopic erosive gastritis. Dig Dis Sci. 2011;56(8):2354–60. Epub 2011/03/31. doi: 10.1007/s10620-011-1681-3 . 21448696

[19] Dixon MF. Gastrointestinal epithelial neoplasia: Vienna revisited. Gut. 2002;51(1):130–1. Epub 2002/06/22. doi: 10.1136/gut.51.1.130 ; PubMed Central PMCID: PMC1773259. 12077106 PMC1773259

[20] Zhao G, Xue M, Hu Y, Lai S, Chen S, Wang L. How Commonly Is the Diagnosis of Gastric Low Grade Dysplasia Upgraded following Endoscopic Resection? A Meta-Analysis. PLoS One. 2015;10(7):e0132699. Epub 2015/07/17. doi: 10.1371/journal.pone.0132699 ; PubMed Central PMCID: PMC4504521. 26182344 PMC4504521

[21] Kato M. Diagnosis and therapies for gastric non-invasive neoplasia. World J Gastroenterol. 2015;21(44):12513–8. Epub 2015/12/08. doi: 10.3748/wjg.v21.i44.12513 ; PubMed Central PMCID: PMC4658607. 26640329 PMC4658607

[22] Akbari M, Kardeh B, Tabrizi R, Ahmadizar F, Lankarani KB. Incidence Rate of Gastric Cancer Adenocarcinoma in Patients With Gastric Dysplasia: A Systematic Review and Meta-Analysis. J Clin Gastroenterol. 2019;53(10):703–10. Epub 2019/08/16. doi: 10.1097/MCG.0000000000001257 . 31415022

[23] Goldstein NS, Lewin KJ. Gastric epithelial dysplasia and adenoma: historical review and histological criteria for grading. Hum Pathol. 1997;28(2):127–33. Epub 1997/02/01. doi: 10.1016/s0046-8177(97)90095-2 . 9023391

[24] Kato I, Tominaga S, Ito Y, Kobayashi S, Yoshii Y, Matsuura A, et al. Atrophic gastritis and stomach cancer risk: cross-sectional analyses. Jpn J Cancer Res. 1992;83(10):1041–6. Epub 1992/10/01. doi: 10.1111/j.1349-7006.1992.tb02719.x ; PubMed Central PMCID: PMC5918674. 1452455 PMC5918674

[25] Kato I, Tominaga S, Ito Y, Kobayashi S, Yoshii Y, Matsuura A, et al. A prospective study of atrophic gastritis and stomach cancer risk. Jpn J Cancer Res. 1992;83(11):1137–42. Epub 1992/11/01. doi: 10.1111/j.1349-7006.1992.tb02736.x ; PubMed Central PMCID: PMC5918704. 1483928 PMC5918704

[26] Correa P, Piazuelo MB. The gastric precancerous cascade. J Dig Dis. 2012;13(1):2–9. Epub 2011/12/23. doi: 10.1111/j.1751-2980.2011.00550.x ; PubMed Central PMCID: PMC3404600. 22188910 PMC3404600

[27] Bisschops R, Areia M, Coron E, Dobru D, Kaskas B, Kuvaev R, et al. Performance measures for upper gastrointestinal endoscopy: a European Society of Gastrointestinal Endoscopy (ESGE) Quality Improvement Initiative. Endoscopy. 2016;48(9):843–64. Epub 2016/08/23. doi: 10.1055/s-0042-113128 . 27548885

[28] Park JM, Huo SM, Lee HH, Lee BI, Song HJ, Choi MG. Longer Observation Time Increases Proportion of Neoplasms Detected by Esophagogastroduodenoscopy. Gastroenterology. 2017;153(2):460–9 e1. Epub 2017/05/16. doi: 10.1053/j.gastro.2017.05.009 . 28501581

[29] Shibagaki K, Amano Y, Ishimura N, Taniguchi H, Fujita H, Adachi S, et al. Diagnostic accuracy of magnification endoscopy with acetic acid enhancement and narrow-band imaging in gastric mucosal neoplasms. Endoscopy. 2016;48(1):16–25. Epub 2015/07/15. doi: 10.1055/s-0034-1392542 . 26158242

[30] Kim JM, Sohn JH, Cho MY, Kim WH, Chang HK, Jung ES, et al. Inter-observer Reproducibility in the Pathologic Diagnosis of Gastric Intraepithelial Neoplasia and Early Carcinoma in Endoscopic Submucosal Dissection Specimens: A Multi-center Study. Cancer Res Treat. 2019;51(4):1568–77. Epub 2019/04/12. doi: 10.4143/crt.2019.019 ; PubMed Central PMCID: PMC6790834. 30971066 PMC6790834

